# Medium-chain fatty acid-rich oils with varying free fatty acid levels as alternatives to soybean oil in broiler starter diets

**DOI:** 10.1016/j.psj.2026.107083

**Published:** 2026-05-07

**Authors:** M. Espinosa-de-los-Monteros-Peñafiel, L. Castillejos, R. Sala, A. Tres, M.D. Soler, A.C. Barroeta

**Affiliations:** a Animal Nutrition and Welfare Service (SNiBA), Department of Animal and Food Sciences, Universitat Autònoma de Barcelona, Bellaterra 08193, Spain; b Departament de Nutrició, Ciències de l’Alimentació i Gastronomia, Universitat de Barcelona. Santa Coloma de Gramenet 08921, Spain; c Institut de Recerca en Nutrició i Seguretat Alimentària, Universitat de Barcelona. Santa Coloma de Gramenet 08921, Spain; d AviFeed Science, Department of Animal Production and Health, Faculty of Veterinary Medicine, Universidad CEU Cardenal Herrera − CEU Universities, Alfara del Patriarca, Valencia 46115, Spain

**Keywords:** Medium-chain fatty acids, Free fatty acids, Palm kernel oil, Black soldier fly larvae oil, Palm kernel fatty acid distillate

## Abstract

This study evaluated the effects of a 4% dietary supplementation with oils rich in medium-chain fatty acids (**MCFA**), varying in free fatty acids (**FFA**) content, on fatty acid (**FA**) digestibility and intestinal health in starter broilers. A total of 512 one-day-old female broilers (Ross 308) were fed four experimental diets (8 replicates/treatment), replacing soybean oil (**SO**) with black soldier fly larvae oil (**BSFO**), palm kernel oil (**PKO**), or palm kernel FA distillate from physical refining (**PKFAD**). Broilers fed SO, BSFO, or PKO showed higher body weight, daily gain, feed intake, and apparent metabolizable energy (**AME**) intake on day 21 than those fed PKFAD (*P* < 0.001). Diets with BSFO and PKO oils, rich in MCFA (36–49%) and low in FFA (<3%), maintained ileal total FA digestibility comparable to the SO diet (*P* < 0.001), achieving up to 89% of C12:0 absorption at the distal ileum, of which over 92% occurred in the jejunum. In contrast, PKFAD showed the lowest digestibility coefficients for most FA, likely due to the high long-chain saturated FA (**LCSFA**; 38.2%) and FFA (68.2%) contents of the added oil. Intestinal morphology and ileal microbial counts were unaffected, but cecal propionate concentrations were higher in BSFO and PKO compared to PKFAD, indicating enhanced microbial fermentation. These results support including BSFO and PKO at 4% in broiler starter diets as alternative energy sources, promoting rapid and efficient MCFA absorption whereas maintaining growth and gut health. In contrast, high FFA levels in PKFAD limited MCFA absorption and negatively affected AME intake and performance.

## Introduction

Dietary oils are critical ingredients of broiler diets due to their high energy contribution and their role in supplying essential fatty acids (**FA**; Ravindran et al., 2016; Abdulla et al., 2019). The rising cost and fluctuating availability of conventional fat sources, such as soybean and palm oils, highlight the need to explore alternative fat sources that promote resource efficiency and the reuse of agro-industrial by-products, thereby reducing environmental impact.

Medium-chain fatty acids (**MCFA**; C6:0–C12:0) have gained interest in poultry nutrition, as they are highly digestible, provide rapidly available energy, and have been associated with antimicrobial effects that could contribute to intestinal health and broiler performance during the early life (Baltić et al., 2018; Ee et al., 2024). Information regarding the nutritional role of free MCFA is still scarce, although it has been suggested that their utilization may be influenced by the degree of esterification (Zeitz et al., 2015). Conversely, it is well established that the digestibility of long-chain saturated fatty acids (**LCSFA**; ≥C14) is compromised in young broilers, particularly when present in free form (Wiseman et al., 1998; Ravindran et al., 2016; Rodriguez-Sanchez et al., 2019a; Jimenez-Moya et al., 2021a).

Palm kernel oil, palm kernel fatty acid distillate, and black soldier fly larvae oil are fat sources rich in MCFA, with lauric acid (C12:0) representing more than 35% of their fatty acid composition. Palm kernel oil and black soldier fly larvae oil are predominantly composed of triacylglycerols (**TAG**), whereas palm kernel fatty acid distillate, a by-product of crude palm kernel oil refining, contains more than 60% free fatty acids (**FFA**; Kareem et al., 2018; Varona et al., 2021a).

Based on this, it is hypothesized that MCFA-rich fat sources could replace conventional oils in starter broiler diets, supporting nutrient absorption, growth performance, and gut health. However, although the use of MCFA as low-dose additives (<1%) has been widely studied, there is limited information regarding their inclusion at higher dietary levels (e.g., >2%), particularly, on its effects on nutrient utilization and gut health in young broilers with immature digestive systems. Additionally, there is a need to evaluate whether these effects would be similar if MCFA are provided as TAG or as FFA, as this might be the case when using fat by-products rich in FFA such as PKFAD (Varona et al., 2021b). Therefore, this study aims to evaluate the impact of incorporating MCFA-rich fat sources with different FFA levels in starter broiler diets on fatty acid digestibility, growth performance, and intestinal health variables, including histomorphology, microbial populations, and cecal fermentation.

## Materials and methods

### Experimental fats

The chemical composition of experimental fats is presented in Table 1. Soybean oil (**SO**), palm kernel oil (**PKO**) and palm kernel fatty acid distillate (**PKFAD**) were supplied by LIPSA (Barcelona, Spain), whereas black soldier fly larvae oil (**BSFO**) was supplied by Protix Ingredients B.V. (Dongen, The Netherlands). All fat samples were analyzed as described by Varona et al. (2021a, 2021b).

**Table 1:** Composition of experimental oils included in the starter diets of broiler chickens.

	Experimental fats^1^			
Item	SO	BSFO	PKO	PKFAD
Fatty acid composition, %				
C6:0	N.D.	N.D.	0.2	0.2
C8:0	N.D.	N.D.	2.6	3.3
C10:0	N.D.	0.80	2.9	3.6
C12:0	N.D.	35.6	43.3	42.7
C14:0	0.1	9.5	17.6	16.7
C16:0	10.8	16.6	9.8	14.7
C16:1 n-7	0.1	2.6	N.D.	<0.1
C18:0	4.5	3.0	2.6	6.7
C18:1 *trans*	0.1	0.1	<0.1	0.1
C18:1 n-9	20.7	15.3	17.6	9.9
C18:1 n-7	1.4	0.4	0.1	0.1
C18:2 n-6	54.0	14.6	2.8	1.9
C18:3 n-3	7.7	1.3	0.1	<0.1
C20:0	0.3	0.1	0.1	0.1
C20:1 n-9	N.D.	0.2	N.D.	<0.1
C22:0	0.3	N.D.	<0.1	N.D.
SFA	16.0	65.6	79.3	87.9
MCFA	N.D.	36.4	49.0	49.7
LCSFA	16.0	29.2	30.2	38.2
MUFA	22.2	18.4	17.8	10.0
PUFA	61.8	15.9	2.9	2.0
(UFA+MCFA)/LCSFA	5.2	2.4	2.3	1.6
PUFA/LCSFA	3.9	0.5	0.1	<0.1
Lipid-class composition, %				
TAG	100.0	99.6	98.2	24.1
DAG	N.D.	0.3	1.3	5.7
MAG	N.D.	N.D.	N.D.	2.0
FFA	N.D.	0.1	0.6	68.2
MIU, %	0.99	1.28	0.62	1.11
Moisture	0.01	0.38	0.03	0.11
Impurities	0.21	0.23	0.26	0.08
Unsaponifiable	0.77	0.67	0.33	0.92

Abbreviations: SO: soybean oil; BSFO: black soldier fly larvae oil; PKO: palm kernel oil; PKFAD: palm kernel fatty acid distillate; SFA: saturated fatty acids; MCFA: medium-chain fatty acids 6-12 C; LCSFA: long-chain saturated fatty acids (≥ C14:0); MUFA: monounsaturated fatty acids; PUFA: polyunsaturated fatty acids; UFA: unsaturated fatty acids; TAG: triglycerides; DAG: diglycerides; MAG: monoglycerides; FFA: free fatty acids; MIU, moisture, impurities and unsaponifiable; N.D.: not detected.

1 All samples were analyzed in duplicate.

### Housing and animals

The study was conducted at the facilities of the *Servei de Granges i Camps Experimentals* (Universitat Autònoma de Barcelona; Bellaterra, Barcelona, Spain). All procedures were approved by the Animal Ethics Committee (CEEAH) of the same institution (reference code: 4006; DMAH 10167) in accordance with European Union Directive 2010/63/EU. A total of 512 newly hatched female broiler chickens (Ross 308) were obtained from a commercial hatchery (Pondex SAU; Lleida, Spain). Upon arrival, the birds were wing-banded and individually weighed (42.33 g ± 2.63 g) and were randomly distributed in 32 environmentally controlled metabolic cages and were assigned to 4 dietary treatments, with 8 replicates per treatment (16 animals/replicate). Each cage was equipped with a wire-floor and a tray for excreta collection. Chickens had ad libitum access to feed and fresh water throughout the study and were maintained under environmental conditions recommended by the Ross 308 management handbook (Aviagen, 2022). The animals and housing facilities were inspected a minimum of four times daily.

### Experimental design and diets

Birds were fed a starter diet based on wheat and soybean meal (Table 2), in mash form from day 0 to day 21. This diet was formulated in accordance with the nutritional requirements established by FEDNA (2018) whereas minimizing the basal fat content.

**Table 2:** Ingredient composition of the starter diet (as-fed basis) for broiler chickens.

Ingredients	%
Wheat	52.41
Soybean meal 47%	25.78
Barley	8.00
Wheat middlings	5.45
Experimental oil^1^	4.00
Calcium carbonate	1.22
Dicalcium phosphate	1.17
L-Lysine·HCl	0.44
Sodium chloride	0.36
DL-Methionine	0.34
Vit-Min. premix^2^	0.30
L-Threonine	0.22
L-Valine	0.15
Sodium bicarbonate	0.10
Choline	0.06

1 Soybean oil, Black Soldier Fly larvae oil, Palm Kernel oil, or Palm Kernel Fatty Acid Distillate.

2 Provides, per kg of feed: Vitamin A (retinyl acetate), 9,000 IU; vitamin D3, 3,200 IU; vitamin E (alpha-tocopherol acetate), 60 IU; vitamin B12, 15.0 μg; vitamin B6, 3.0 mg; vitamin K3, 1.99 mg; vitamin B1, 1.99 mg; vitamin B2, 5.01 mg; niacin, 39.99 mg; folic acid, 0.999 mg; biotin, 99 μg; Fe, 50.01 mg; Mn, 99.99 mg; Se, 0.498 mg; Zn (hydroxylated methionine zinc chelate), 39.99 mg; Zn (zinc sulfate monohydrate) 39.99 mg; Cu, 15.24 mg; I, 0.999 mg: 6-Phytase 4a19, 750 FTU; endo-1,4-beta-xylanase, 24,000 BXU.

Experimental diets were prepared by supplementing the basal diet with 4% of different fat sources: BSFO (36.4% MCFA and 0.1% FFA), PKO (49.0% MCFA and 0.6% FFA), or PKFAD (by-product of PKO physical refining; 49.7% MCFA and 68.2% FFA). A diet with SO (61.8% polyunsaturated FA (**PUFA**); N.D.% MCFA and N.D.% FFA) was used as control. All diets included 50 mg/kg of Ytterbium oxide (**Yb_2_O_3_**; 246999 Sigma-Aldrich Chemical Co.; St. Louis, MO). The basal diet was manufactured at Pinsos Molinet S.A., (Prats de Lluçanès, Barcelona, Spain) and the addition of the experimental fat sources and the inert marker for the preparation of the experimental diets was carried out at the Servei de Granges i Camps Experimentals of the Universitat Autònoma de Barcelona.

### Controls and sampling

Individual body weight (**BW**) and feed intake per cage were recorded on 9 and 21 days of age to calculate the average daily feed intake (**ADFI**), average daily gain (**ADG**), and the feed conversion ratio (**FCR**). Mortality was recorded, and individual weights were taken to adjust these variables. At day 9, samples were collected to conduct digestibility balance and intestinal health assessment (histomorphology, microbiological counts, and volatile fatty acid (**VFA**) analysis).

For all these analyses, 12 birds per cage were euthanized by cervical dislocation. The entire jejunum (from the distal insertion of the duodenal mesentery to the junction with Meckel's diverticulum) and ileum (from the junction with Meckel's diverticulum to a point 1 cm proximal to the ileocecal junction) were removed and each divided into two equal segments. Only the distal segments of both jejunum and ileum were used for sampling digestive content and tissue.

For digestibility determination (6 replicates per treatment), digesta from the distal segments of both jejunum and ileum of 10 birds per cage were pooled, homogenized, and stored at −20 °C. Samples were subsequently freeze-dried, ground to pass through a 0.5 mm sieve, and stored at 4 °C until analysis.

For intestinal health measurements (8 replicates per treatment), digesta from the distal jejunum and ileum of two birds per cage were pooled and immediately placed on ice for microbiological analysis by classical plate counting of selected bacterial groups. One bird per cage was used for VFA analyses and histomorphology evaluation. A 2 cm section from the middle of the distal jejunum and ileum was collected, opened longitudinally, rinsed with PBS, and fixed by immersion in a 4% buffered formaldehyde solution. Moreover, individual cecal content was collected and stored at −80 °C for VFA determination.

### Chemical analysis

Experimental fats underwent duplicate analysis to determine the FA composition, acid value (FFA content), and the moisture, impurities, and unsaponifiable matter content, following the methods outlined by Varona et al. (2021b). The diets were subjected to analytical determinations following the methods outlined by the AOAC International (2025), including measurements for dry matter (934.01), ash (942.05), crude protein (968.06), crude fat (2003.05), and crude fiber (962.09). Gross energy of experimental feeds and excreta samples were assessed using an adiabatic calorimeter (IKA C-4000, Janke-Kunkel; Staufen, Germany). Concentrations of Yb_2_O_3_ were determined in experimental diets, digestive contents, and excreta samples according to the AOAC 984.27 method using an optical emission spectrometer ICP-OES 5900 (Agilent Technologies, Santa Clara, California, USA).

For the lipid class composition, fat was extracted from diets as described by Jimenez-Moya et al. (2021a, 2021b). Lipid classes were determined by GC-MS using an Agilent 6890N gas chromatograph coupled to an Agilent 5973 Network mass selective detector (Agilent Technologies, Santa Clara, CA), with helium as carrier gas at 1.5 ml/min constant flow. Injection was conducted in splitless mode at 370°C. Separation was conducted on a Rtx-65TG column (30 m × 0.25 mm × 0.1 μm, Restek, Bellefonte, PA, USA), and oven was programmed as follows: 80 °C, to 320 °C at 10 °C/min, hold 0.1 min; to 350 °C at 2.5 °C/min; hold 10 min. Transfer line temperature was 350 °C, and ionization energy was 70 eV. Acquisition in complete scanning mode was conducted in the range 40–660 m/z. Compound identification was conducted according to the NIST 2.0 library (National Institute of Standards and Technology).

The FA content in the feed, digestive content, and excreta was determined following the method described by Sukhija and Palmquist (1988). This analytical procedure involves direct transesterification, which includes lipid extraction and FA methylation. Samples were incubated at 70 °C with methanolic hydrochloric acid (a mixture of methanol and acetyl chloride) for methylation. Nonadecanoic acid (C19:0; Sigma-Aldrich Chemical Co.; St. Louis, MO) was added as an internal standard before methylation. Following extraction and methylation, potassium carbonate and toluene were added to facilitate the separation of the organic layer. The final extract was injected into a gas chromatograph (HP6890, Agilent Technologies; Waldbronn, Germany) equipped with a flame ionization detector (**FID**), following the conditions described previously by Cortinas et al. (2004). The FA methyl esters were identified by matching their retention times with those of their respective standards (Supelco 37 component FAME Mix, Sigma-Aldrich Co.). Quantification of individual FA was performed using external calibration curves prepared from the same FA methyl esters standards. The macronutrient and FA composition of experimental diets are described in Table 3.

**Table 3:** Macronutrient and energy content, fatty acid and lipid class composition of the diets fed to broiler chickens.

	**Starter diets**			
Item^1^	SO	BSFO	PKO	PKFAD
Macronutrient and energy content, %				
Dry matter	89.96	90.48	90.12	90.14
Crude protein	19.49	19.00	19.21	19.04
SID Methionine*	0.58	0.58	0.58	0.58
SID Lysine*	1.15	1.15	1.15	1.15
Ether extract	5.33	5.69	5.50	5.41
Crude fiber	4.02	4.21	3.68	4.23
Ash	4.90	5.11	4.84	4.71
Calcium*	0.98	0.98	0.98	0.98
Phosphorus*	0.59	0.59	0.59	0.59
Digestible phosphorus*	0.42	0.42	0.42	0.42
Chloride*	0.35	0.35	0.35	0.35
Sodium*	0.20	0.20	0.20	0.20
Gross energy, kcal/kg	4,125	4,113	4,106	4,113
Fatty acid composition, %				
C8:0	N.D.	N.D.	2.0	2.3
C10:0	N.D.	0.5	1.9	2.2
C12:0	1.8	24.4	28.7	28.0
C14:0	N.D.	5.6	9.5	9.0
C16:0	13.6	16.5	12.5	15.1
C18:0	4.2	3.1	3.0	5.2
C18:1 n-9	19.2	15.4	16.4	12.2
C18:1 n-7	1.4	0.7	0.5	0.5
C18:2 n-6	52.6	29.7	23.5	23.3
C18:3 n-3	6.4	2.7	2.0	2.1
C20:0	0.4	N.D.	N.D.	N.D.
C20:1 n-9	0.3	N.D.	N.D.	N.D.
C22:0	0.1	N.D.	N.D.	N.D.
Minor fatty acids %	N.D.	1.36	N.D.	0.1
SFA	20.2	50.0	57.5	61.8
MCFA	1.8	25.0	32.5	32.5
LCSFA	18.4	25.0	25.0	29.3
MUFA	20.9	17.6	17.0	12.8
PUFA	58.9	32.4	25.5	25.4
(UFA+MCFA)/LCSFA	4.4	3.0	3.0	2.4
PUFA/LCSFA	3.2	1.3	1.0	0.9
**Lipid-class composition, %**				
TAG	86.0	87.7	88.8	28.8
DAG	4.1	2.8	3.1	4.6
MAG	N.D.	N.D.	N.D.	0.5
FFA	9.9	9.5	8.1	66.1

Abbreviations: SO: soybean oil 4%; BSFO: black soldier fly larvae oil 4%; PKO: palm kernel oil 4%; PKFAD: palm kernel fatty acid distillate 4%; SFA: saturated fatty acids; MUFA: monounsaturated fatty acids; PUFA: polyunsaturated fatty acids; UFA: unsaturated fatty acids; MCFA: medium-chain fatty acids (6-12 C); LCSFA: long-chain saturated fatty acids (≥14:0 C); TAG: triglycerides; DAG: diglycerides; MAG: monoglycerides; FFA: free fatty acids N.D.: not detected.

⁎ Calculated composition.

1 All samples were analyzed in duplicate.

Samples used for the histomorphological study were processed according to the standard paraffin inclusion procedure. Sections with a thickness of 4 μm were then obtained, stained with Hematoxylin-Eosin, and digitized using a LEICA SCN400 scanner (Leica Microsystems IR GmbH, Wetzlar, Germany). The ViewPoint Light image analysis program (Precipoint, Freising, Germany) was utilized to measure villus height (**Vh**; from the villus-crypt junction to the apical end of the villus) and crypt depth (**Cd**; from the base of the crypt to the villus-crypt junction). Thirty measurements of Vh and Cd were taken for each animal to calculate the Vh/Cd ratio.

For microbiological counts, samples of jejunal and ileal contents were serially diluted (1:10) in lactated Ringer’s solution (Sigma-Aldrich Co.; St. Louis, MO, USA) and plated onto de Man, Rogosa and Sharpe (MRS) agar for lactic acid bacteria enumeration and onto MacConkey agar for Enterobacteriaceae counts.

The plates were then incubated for 48 h. To quantitatively assess the total lactic acid bacteria, the culture plates were placed in incubators set to aerobic, microaerophilic (with 5% CO_2_), and anaerobic conditions at 37 °C. Additionally, to detect the presence of Enterobacteriaceae, cultures were inoculated under aerobic conditions at both 37 °C and 42 °C. *Escherichia coli* and coliform bacteria were isolated among all enterobacteria and subsequently re-incubated under anaerobic conditions at 37°C for 48 h. All data are presented as Colony Forming Units per gram (cfu/g).

Finally, for VFA analysis, 250 mg of cecal content were gathered into an Eppendorf tube, and 1 mL of a 2 mM calcium solution was added. Samples were homogenized using a vortex mixer and centrifuged (15,000 × g) at 7 °C for 10 min. The resulting supernatant was collected and acidified with 5% phosphoric acid prior to analysis. The VFA were analyzed by gas chromatography with flame ionization detection (GC–FID), following the methodology described by Richardson et al. (1989) and modified by Jensen et al. (1995).

### Calculations

The apparent digestibility of FA (X) was calculated as follows:

%ApparentdigestibilityofX={1−[(Xf/Mf)/(Xd/Md)]}×100

where X represents the level of a specific FA found in feces or digestive content (f), and in diet (d), whereas M indicates the concentration of the inert marker in feces or digestive content (f) and in diet (d). The apparent metabolizable energy (**AME**) intake was calculated as dietary AME multiplied by average daily feed intake (g/bird per day), with AME calculated as the product of feed gross energy and the apparent metabolizable energy coefficient.

### Statistical analysis

Data normality and variance homogeneity were assessed using the Shapiro-Wilk tests in the R statistical package (version 4.2.2). All statistical analyses were conducted using one-way ANOVA in R software, with diet considered as the primary factor for all variables. The experimental unit was the cage, with 6 replicates per treatment for digestibility and 8 replicates for intestinal health assessment, and differences between means were evaluated using Tukey’s adjustment for multiple comparisons. The results presented in the tables represent least squares means. Significance was established at *P* ≤ 0.05 for all statistical analyses.

## Results

### Characterization of experimental oils and diets

The composition of the experimental oils is presented in Table 1. Regarding the FA composition, SO had the highest PUFA content (61.8%), mainly linoleic acid (C18:2 n-6), and the lowest content of saturated fatty acids (**SFA**; 16.0%). In the case of BSFO, PKO, and PKFAD, they presented >65% SFA due to their high MCFA content (>35%), with lauric acid (C12:0) as the main FA. The BSFO had lower lauric acid (35.6%) and higher PUFA (15.9%) levels than palm kernel derived oils (C12:0 ranging from 42.7–43.3%, and PUFA ranging from 2.0–2.9%). Among all the experimental oils, PKFAD had the highest LCSFA (SFA with ≥14C; 38.2%) and FFA content (PKFAD: 68.2%; SO: N.D.%; BSFO: 0.1%; PKO: 0.6%). The total moisture, impurities, and unsaponifiable content (**MIU**) of the oils utilized in this study was below 1.3%.

The composition of experimental diets is shown in Table 3. Gross energy and all macronutrient content values were similar among dietary treatments. The FA composition reflected that of the corresponding experimental oils, with the diets containing BSFO and palm kernel derived fats (PKO and PKFAD) being richer in MCFA, whereas the SO diet was richer in PUFA. In terms of dietary lipid-class composition, triacylglycerols were the main lipid-class in SO, BSFO, and PKO diets (86.0–88.8%), whereas the PKFAD diet contained a much lower TAG proportion (28.8%) and was dominated by FFA (66.1%).

### Growth performance

The effects of the SO, BSFO, PKO, and PKFAD diets on growth performance are shown in Table 4. Animals fed the SO, BSFO, and PKO diets had significantly higher BW on days 9 and 21, as well as greater ADFI and ADG, compared to those on the PKFAD diet (*P* < 0.001). At the end of the trial, the FCR for the SO diet (1.24) was similar to that of the BSFO diet (1.28) and lower than both the PKO (1.32) and PKFAD (1.35) diets (*P* < 0.001). In addition, AME intake differed among treatments (*P* < 0.001), being higher in broilers fed SO, BSFO, and PKO compared with PKFAD.

**Table 4:** Effect of the experimental diets on growth performance and AME intake in broiler chickens (0 to 21 days).

	Dietary treatments					
Item^1^	SO	BSFO	PKO	PKFAD	SEM	*P*-value
BW 0 days, g	42.4	42.4	42.4	42.4	0.23	0.999
BW 9 days, g	228^a^	234^a^	232^a^	207^b^	2.13	<0.001
BW 21 days, g	881^a^	864^a^^b^	835^b^	766^c^	11.2	<0.001
From 0 to 21 days						
ADFI, g/d	51.8^a^	53.0^a^	52.3^a^	48.2^b^	0.66	<0.001
ADG, g/d	41.7^a^	41.3^a^	40.4^a^	36.3^b^	0.60	<0.001
FCR, g/g	1.24^c^	1.28^b^^c^	1.32^a^^b^	1.35^a^	0.016	0.001
AME intake, kcal/b/d	159^a^	155^a^	158^a^	143^b^	2.64	<0.001

Abbreviations: SO: soybean oil; BSFO: black soldier fly larvae oil; PKO: palm kernel oil; PKFAD: palm kernel fatty acid distillate; SEM: standard error of the mean; BW: body weight; ADFI: average daily feed intake; ADG: average daily gain; FCR: feed conversion ratio.

1 Values of ADFI and ADG are expressed on as-fed basis.

a-c Values within a row with different superscripts differ significantly at *P≤* 0.05 according to one-way ANOVA (*n* = 8 replicates per treatment).

### Digestibility balance

The results of apparent FA digestibility in the distal jejunum, distal ileum, and total tract are presented in Table 5. Regarding FA, SFA were divided into medium-chain (MCFA: C6:0, C8:0, C10:0 and C12:0) and long-chain saturated FA (LCSFA: predominantly C14:0, C16:0 and C18:0). Unsaturated FA (**UFA**) included monounsaturated FA (**MUFA**) were mainly represented by oleic acid (C18:1 n-9), whereas PUFA were primarily linoleic (C18:2 n-6) and alpha-linolenic (C18:3 n-3) acids. Broilers fed MCFA-rich diets (PKO, BSFO, and PKFAD) showed higher MCFA digestibility in the distal jejunum and distal ileum compared to SO (*P* < 0.001), particularly for lauric acid (C12:0). At the excreta level, PKO and BSFO maintained higher MCFA digestibility, whereas PKFAD and SO showed significantly lower values (*P* *<* 0.001). In contrast, PUFA digestibility (mainly C18:2 n-6 and C18:3 n-3) was significantly higher in the SO group across all intestinal segments, including excreta compared to BSFO, PKO, and PKFAD (*P* < 0.002). At distal ileal and excreta levels, no significant differences were found in total FA (**TFA**), MUFA, and LCSFA digestibility between SO, BSFO, and PKO. However, in the jejunum, birds fed BSFO showed lower LCSFA digestibility than those fed PKO (*P* = 0.016), as well as lower digestibility of TFA (*P* = 0.005) and MUFA (*P* = 0.007) compared to SO. Broilers fed PKFAD consistently showed the lowest digestibility values for most FA from C12:0 onward, particularly at the jejunal level. When compared with SO, the digestibility of TFA and MUFA was consistently lower in the PKFAD group across all segments, including excreta (*P* < 0.05). Compared to PKO, PKFAD showed significantly lower MCFA digestibility across both intestinal segments and in the excreta (*P* < 0.001), as well as lower LCSFA digestibility at the jejunum (*P* < 0.01), with values that approached significance at the ileum (*P* = 0.055) and in the excreta (*P* = 0.087). In relation to BSFO, PKFAD showed lower MCFA digestibility in the excreta and lower C14:0 digestibility at the ileum and in the excreta (*P* < 0.05).

**Table 5:** Effect of experimental diets on apparent fatty acid digestibility at the distal jejunum, distal ileum, and total tract in broiler chickens at 9 days of age.

	Dietary treatments^1^					
Item	SO	BSFO	PKO	PKFAD	SEM	*P*-value
**Distal jejunum**						
C8:0	N.D.	N.D.	100.0	98.7	0.58	0.145
C10:0	N.D.	100.0^a^	95.4^b^	92.1^b^	0.91	<0.001
C12:0	60.0^c^	79.0^a^^b^	84.9^a^	75.9^b^	1.88	<0.001
C14:0	N.D.	68.8^a^^b^	77.3^a^	61.6^b^	3.04	0.008
C16:0	52.4	46.1	57.3	43.1	3.74	0.061
C18:0	41.7	24.8	43.4	32.2	5.25	0.050
C18:1 n-9	67.9	55.2	66.9	57.9	3.54	0.027
C18:2 n-6	81.6^a^	65.4^b^	68.1^b^	64.0^b^	2.95	0.002
C18:3 n-3	87.2^a^	72.7^b^	73.4^b^	71.1^b^	2.34	<0.001
TFA	72.7^a^	62.4^b^^c^	71.7^a^^b^	62.2^c^	2.4	0.005
MCFA	60.0^c^	79.3^a^^b^	86.1^a^	78.6^b^	1.73	<0.001
LCSFA	49.3^a^^b^	47.2^b^	62.2^a^	46.2^b^	3.54	0.016
MUFA	67.6^a^	54.0^b^	64.7^a^^b^	52.2^b^	3.31	0.007
PUFA	82.2^a^	65.7^b^	67.4^b^	64.6^b^	2.83	0.001
**Distal ileum**						
C8:0	N.D.	N.D.	98.0	95.9	0.75	0.074
C10:0	N.D.	100.0^a^	97.8^b^	95.4^c^	0.63	<0.001
C12:0	62.2^b^	89.0^a^	89.5^a^	83.2^a^	2.36	<0.001
C14:0	N.D.	81.6^a^	83.1^a^	69.4^b^	2.64	0.004
C16:0	63.9	61.6	63.1	52.2	3.68	0.087
C18:0	51.6	47.9	57.2	42.5	5.35	0.290
C18:1 n-9	77.0^a^	73.0^a^^b^	72.4^a^^b^	65.2^b^	2.65	0.037
C18:2 n-6	88.7^a^	75.5^b^	72.8^b^	72.3^b^	2.23	<0.001
C18:3 n-3	92.8^a^	80.6^b^	78.3^b^	77.4^b^	1.49	<0.001
TFA	79.9^a^	76.4^a^^b^	77.5^a^^b^	70.5^b^	2.21	0.042
MCFA	61.0^c^	89.2^a^^b^	90.5^a^	85.0^b^	1.30	<0.001
LCSFA	60.9	63.1	68.8	55.0	3.61	0.055
MUFA	76.7^a^	73.1^a^^b^	71.2^a^^b^	63.8^b^	2.69	0.020
PUFA	89.3^a^	78.2^b^	72.2^b^	73.4^b^	2.04	<0.001
**Excreta**						
C8:0	N.D.	N.D.	100.0	99.63	0.25	0.302
C10:0	N.D.	100.0^a^	100.0^a^	95.51^b^	0.074	<0.001
C12:0	87.2^b^	91.7^a^	92.0^a^	86.5^b^	0.90	<0.001
C14:0	N.D.	84.0^a^	84.4^a^	73.1^b^	1.9	<0.001
C16:0	71.8^a^	66.1^a^^b^	66.3^a^^b^	57.6^b^	2.61	0.007
C18:0	67.2^a^	51.4^b^	56.0^a^^b^	48.8^b^	3.72	0.008
C18:1 n-9	79.4^a^	72.4^a^^b^	75.5^a^^b^	67.8^b^	2.29	0.010
C18:2 n-6	87.3^a^	73.5^b^	74.3^b^	74.2^b^	2.48	0.001
C18:3 n-3	91.5^a^	78.2^b^	78.2^b^	81.5^b^	2.06	<0.001
TFA	81.7^a^	76.4^a^^b^	79.6^a^^b^	73.5^b^	2.04	0.041
MCFA	87.2^b^	91.9^a^	92.9^a^	88.1^b^	0.83	0.001
LCSFA	67.6	67.6	71.0	59.8	3.25	0.087
MUFA	78.8^a^	72.2^a^^b^	74.2^a^^b^	65.5^b^	2.36	0.004
PUFA	87.8^a^	73.9^b^	74.7^b^	74.6^b^	2.45	0.001

1 Values are pooled means of 6 replicates from chickens (10 birds per replicate) fed diets supplemented with 4% of: soybean oil (SO), black soldier fly larvae oil (BSFO), palm kernel oil (PKO), and palm fatty acid distillate (PKFAD). TFA: total fatty acids, MUFA: monounsaturated fatty acids, PUFA: polyunsaturated fatty acids, MCFA: medium chain fatty acids, LCSFA: long chain saturated fatty acids. SEM: standard error of the mean.

a-c Values within a row with different superscripts differ significantly at *P* ≤ 0.05. N.D.: not detected.

**Fig. 1:**
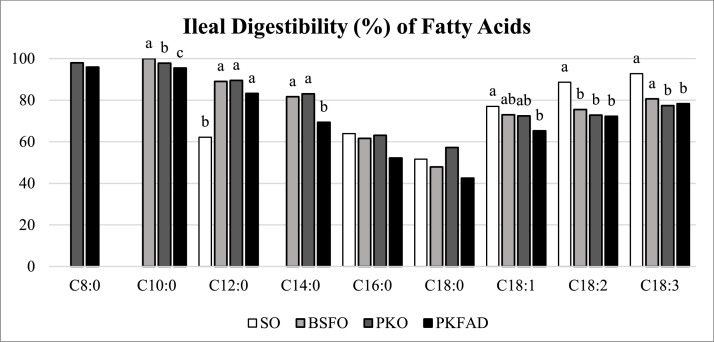
Apparent digestibility (%) of individual fatty acids in the distal ileum of 9-day-old broiler chickens fed different diets with 4% of: soybean oil (SO), black soldier fly larvae oil (BSFO), palm kernel oil (PKO), or palm fatty acid distillate (PKFAD). Values are means of 6 replicates per diet (10 chickens per replicate). Within each fatty acid, bars with different superscripts (a-c) differ significantly (*P<* 0.01). See Table 5 for detailed *P* values.

The digestibility of the individual FA in ileum is shown in Fig. 1. Although jejunum remained a key site of absorption, digestibility at distal ileum was chosen as it reflects the final phase of fat absorption. Among SFA, MCFA showed the highest digestibility, particularly C12:0 showed values around 89–90% in birds fed crude oils rich in MCFA (BSFO and PKO). These values were similar or even higher than those observed for C18:2 n-6 in the conventional oil (SO, 88.7%). In contrast, LCSFA C16:0 and C18:0 consistently presented the lowest digestibility across all treatments with the most limited absorption observed in broilers receiving the PKFAD diet (C16:0: 52.2%; C18:0: 42.5%), which contained high levels of both FFA and LCSFA. Although classified as a LCSFA, C14:0 is utilized more efficiently than C16:0 or C18:0 and it showed higher digestibility values in broilers fed crude oils rich in MCFA (BSFO: 81.6% and PKO: 83.1%) than those receiving PKFAD (69.4%).

**Fig. 2:**
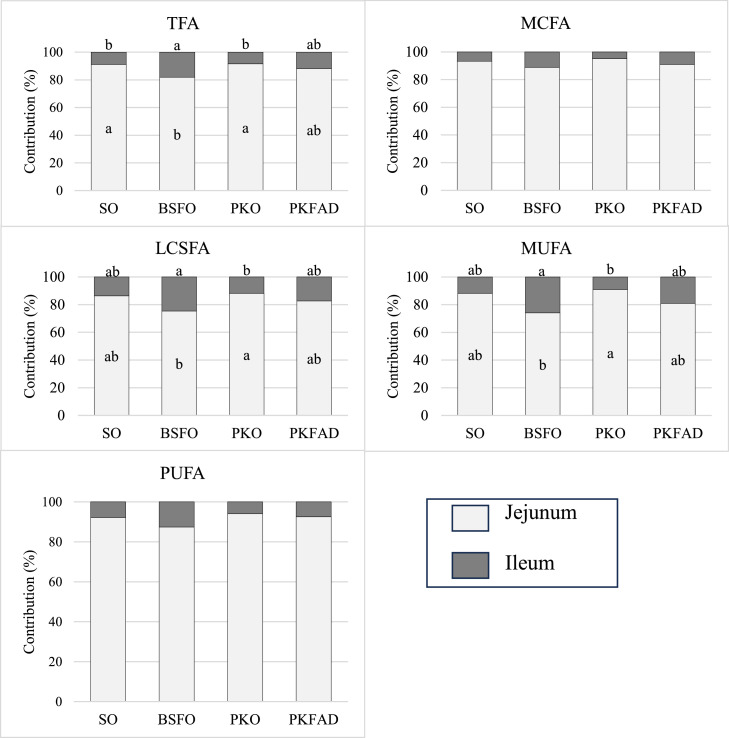
Contribution of the jejunum and ileum to the apparent digestibility of FA, expressed as a percentage of total absorption at distal jejunum level, in 9-day-old broiler chickens fed diets with 4% of: soybean oil (SO), black soldier fly larvae oil (BSFO), palm kernel oil (PKO), or palm fatty acid distillate (PKFAD). Values are means of 6 replicates per diet (10 chickens per replicate). Within each treatment, different superscript letters (a–c) indicate significant differences between intestinal segments (*P<* 0.01) according to one-way ANOVA.

To assess the relative contribution of the jejunum and ileum to FA absorption, values from each segment were calculated as a proportion of total ileal absorption (Fig. 2), although no significant absorption occurs beyond the ileum. The contribution of the jejunum to TFA absorption ranged from 82 to 92% across all dietary treatments, confirming it as the principal site of uptake within the gastrointestinal tract (*P* < 0.001).

### Histomorphology of jejunum and ileum

The influence of the experimental treatments on the histomorphological variables of the distal jejunum and distal ileum is shown in Table 6. In the jejunum, no statistically significant differences were observed among the dietary treatments (*P* > 0.05). However, villus height in the jejunum approached significance (*P* = 0.069), with lower values in broilers fed the PKFAD diet compared with the other treatments. In the ileum, significantly lower villus height was observed in PKFAD compared to PKO (*P* = 0.047), but no significant differences in Cd and Vh/Cd ratio among the experimental diets (*P* > 0.05) were obtained.

**Table 6:** Effect of the experimental diets on histomorphology of the distal jejunum and distal ileum in broiler chickens at 9 days of age.

	Dietary treatments^1^					
	SO	BSFO	PKO	PKFAD	SEM	*P*-value
**Jejunum**						
Vh, μm	661	685	672	594	24.8	0.069
Cd, μm	165	163	172	163	7.18	0.763
Vh/Cd ratio	4.06	4.23	3.95	3.70	0.23	0.410
**Ileum**						
Vh, μm	406^a^^b^	401^a^^b^	427^a^	377^b^	12.2	0.047
Cd, μm	167	164	171	157	4.72	0.251
Vh/Cd ratio	2.51	2.39	2.51	2.40	0.09	0.658

1 Values are pooled means of 8 replicates per diet (2 chickens per replicate) from chickens fed diets supplemented with 4% of soybean oil (SO), black soldier fly larvae oil (BSFO), palm kernel oil (PKO), and palm fatty acid distillate (PKFAD). Vh: Villus height, Cd: crypt depth. SEM: standard error of the mean.

a-c Values within a row with different superscripts differ significantly at *P* ≤ 0.05 according to one-way ANOVA.

### Microbiological counting

The effect of the experimental treatments on microbiological counts is presented in Table 7. Although no statistically significant differences (*P* > 0.05) were observed among the dietary treatments in both the distal jejunum and distal ileum of young broiler chickens, the abundance of Enterobacteriaceae (*P* = 0.070), total coliforms (*P* = 0.075) and E. coli (*P* = 0.065) approached significance, with higher counts observed in MCFA-rich fat diets compared with the SO diet.

**Table 7:** Effect of experimental diets on the microbiological counting of the distal jejunum and ileum in broiler chickens at 9 d of age.

	Dietary treatments^1^					
	SO	BSFO	PKO	PKFAD	SEM^1^	*P*-value
**Jejunum**						
*Lactobacillus*, log cfu/g	7.56	7.23	7.66	7.29	0.38	0.788
Enterobacteriaceae, log cfu/g	3.93	4.28	4.09	3.73	0.19	0.242
Total coliforms, log cfu/g	3.88	4.01	4.06	3.60	0.21	0.376
*Escherichia coli*, log cfu/g	3.80	3.68	3.91	3.59	0.20	0.634
*Lactobacillus:*Enterobacteriaceae ratio	1.95	1.66	1.83	1.90	0.17	0.617
**Ileum**						
*Lactobacillus*, log cfu/g	7.78	7.99	7.77	7.79	0.31	0.940
Enterobacteriaceae, log cfu/g	3.84	5.55	4.38	4.88	0.46	0.070
Total coliforms, log cfu/g	3.79	5.49	4.34	4.76	0.46	0.075
*Escherichia coli*, log cfu/g	3.69	5.40	4.30	4.42	0.45	0.065
*Lactobacillus:*Enterobacteriaceae ratio	2.19	1.57	1.77	1.66	0.19	0.103

^a-c^Values within a row with different superscripts differ significantly at *P* ≤ 0.05. N.D.: not detected.

1 Values are means of 8 replicates per diet (2 pooled chickens per replicate) from broilers fed diets supplemented with 4% soybean oil (SO), black soldier fly larvae oil (BSFO), palm kernel oil (PKO), or palm kernel fatty acid distillate (PKFAD). SEM: standard error of the mean.

### Cecal VFA content

The effects of the experimental fats on cecal fermentation are presented in Table 8. Significant differences were observed only in the proportion of total propionate (*P* = 0.006), with birds fed BSFO and PKO diets showing the highest values (ranging from 2.24% to 2.29%), compared to those fed the PKFAD diet (1.82%).

**Table 8:** Effect of experimental diets on fermentation products in the ceca of broiler chickens at 9 d of age.

	Dietary treatments^1^					
Volatile fatty acids	SO	BSFO	PKO	PKFAD	SEM	*P*-value
Total SCFA, μmol/g	96.1	93.4	84.1	87.2	9.01	0.77
Acetate, %	86.3	87.9	86.7	86.8	1.11	0.76
Propionate, %	1.95^a^^b^	2.29^a^	2.24^a^	1.82^b^	0.10	0.006
Butyrate, %	11.31	9.13	9.71	11.14	1.11	0.38
BCFA, %	0.238	0.329	0.463	0.154	0.16	0.54

1 Values are means of 8 replicates per diet (1 animal per replicate) from broilers fed diets supplemented with 4% of soybean oil (SO), black soldier fly larvae oil (BSFO), palm kernel oil (PKO), and palm fatty acid distillate (PKFAD); short chain fatty acids (SCFA); branched chain fatty acids (BCFA). SEM: standard error of the mean.

a-c Values within a row with different superscripts differ significantly at *P* ≤ 0.05 according to one-way ANOVA.

## Discussion

### Growth performance

Although MCFA-rich fats have potential to improve broiler performance (Szabó et al., 2023; Ee et al., 2024), results in the literature remain inconsistent, highlighting the need for further research to clarify their efficacy and underlying mechanisms. The results of the present study align with previous studies in both broilers and young turkeys, where no significant effects on productive performance were observed when SO was partially or completely replaced by BSFO (Schiavone et al., 2017; Kim et al., 2020; Sypniewski et al., 2020; Chen et al., 2022). Additionally, in the present study, when comparing BSFO *vs.* PKO (MCFA-rich fat sources with low FFA), similar performance results were observed throughout the trial. These findings are consistent with Kim et al. (2020), who observed no significant differences in BW, ADFI, ADG, or FCR at 15 days between broilers fed diets containing BSFO or coconut oil, the latter sharing a similar FA profile with PKO. However, in the present study, replacing SO with PKO reduced BW at 21 days and increased the FCR, whereas ADFI and ADG remained unaffected. These differences in performance could be partly explained by the higher PUFA/LCSFA in birds fed SO compared to those fed PKO (SO: 3.2; PKO: 1.0) because the former fatty acids are more readily digested than the latter (Table 5). This finding contrasts with Wang et al. (2015), who reported no significant differences in performance variables in chicks fed 1.5% coconut oil (47.32% MCFA) compared with those fed soybean oil. To the best of our knowledge, few studies have investigated the effects of MCFA-rich fat sources with varying FFA levels at high inclusion rates in young broiler chickens. In the present study, broilers consuming PKFAD showed lower performance, including the worst FCR and reduced ADFI and ADG compared to those fed MCFA-rich fats with low-FFA (BSFO and PKO) and the commonly used oil (SO). This lower performance could be attributed to the elevated levels of FFA (68.2%) and LCSFA (38.2%) in PKFAD oil, a clear difference from SO, BSFO, and PKO oils, which contained FFA levels below 1.0% and LCSFA below 30.5%.

Several studies have shown that high levels of dietary LCSFA and FFA can impair fat utilization in young broilers (Wiseman and Salvador, 1991; Rodriguez-Sanchez et al., 2019a, 2019b; Jimenez-Moya et al., 2021a, 2021b).

Conversely, Józefiak et al. (2014) reported no differences in performance variables between diets containing 2.2% soybean oil and 2.4% PKFAD (50.6% MCFA; FFA content not reported), both included at lower levels than those evaluated in the present study.

Notably, in the present study, diets supplemented with 4% of PKFAD resulted in lower feed intake in starter broilers, compared with PKO, BSFO and SO diets. This reduction is consistent with the results of Palomar et al. (2020), who reported a rejection of diets containing oils with high FFA levels in laying hens, an effect that may depend on the type of fat source rather than on FFA concentration alone. Additionally, the 2.3% caprylic acid (C8:0) in PKFAD may have contributed to reduced feed intake by influencing the satiety center (Cave, 1982; de los Santos et al., 2008; Haynes et al., 2020). The lower feed intake of PKFAD-fed broilers also limited AME intake, thereby restricting metabolizable energy supply and impairing growth performance. In contrast, no differences in feed intake were found among PKO, BSFO and SO diets. Similarly, Smits et al. (2000) reported that dietary supplementation with 8% coconut oil (40.2% MCFA) did not modify broiler feed intake compared to diets containing soybean oil. Thus, MCFA-rich fats with low FFA levels can be used as viable alternatives to soybean oil without compromising broiler growth, whereas high FFA levels may impair feed intake and performance.

### Digestibility balance

Results obtained pointed out differences in digestibility among fat sources, which could be explained by the influence of FA structure, in particular chain length, on intestinal absorption.

The FA were more efficiently absorbed as chain length decreased and unsaturation increased. Whereas previous studies have discussed the effects of unsaturation or esterification status (Tancharoenrat et al., 2014; Wang et al., 2015; Rodriguez-Sanchez et al., 2021; Jimenez-Moya et al., 2021a, 2021b), our findings emphasize the influence of chain length and the combined impact of these factors on FA digestibility. In this study, MCFA showed digestibility values close to those of PUFA, whereas LCSFA such as C16:0 and C18:0 remained consistently less digestible. Consequently, these findings highlight the importance of distinguishing between different types of SFA, particularly MCFA and LCSFA, as they differ in their digestion and absorption mechanisms. This could be explained by the fact that MCFA are more soluble and can cross the intestinal wall more easily than LCSFA (Çenesiz and Çiftci, 2020).

The inclusion of alternative crude fats rich in MCFA and low in FFA (BSFO and PKO) resulted in high levels of dietary TFA and MUFA digestibility at the distal ileum, with values comparable to those obtained in the diet with the conventional oil (SO). The high utilization of dietary FA from SO may be attributed to its low LCSFA content and high PUFA proportion. Conversely, in BSFO and PKO diets, it may be attributed to their elevated C12:0 content (24.4% and 28.7%, respectively) combined with low FFA levels (<3%).

The higher absorption of PUFA and MCFA relative to LCSFA may be explained by their greater hydrophilicity, which enhances diffusion in aqueous environments and interaction with pancreatic lipase (Salvia-Trujillo et al., 2013; Liu et al., 2021). Additionally, PUFA readily form micelles and can facilitate the incorporation of LCSFA into mixed micelles, supporting the absorption of less soluble FA such as C16:0 and C18:0 (Krogdahl, 1985). Although the mechanisms involved in MCFA absorption and post-absorption have been widely studied, some uncertainty remains regarding their solubilization within the intestinal lumen. Certain studies suggest that MCFA, in a manner similar to short-chain fatty acids (**SCFA**, C2:0, C3:0 and C4:0), can be absorbed without micellar solubilization due to their higher solubility and passive diffusion capacity (Christensen et al., 1989; Çenesiz and Çiftci, 2020). Nonetheless, some evidence suggests that micellar incorporation becomes progressively more important as the carbon chain length increases, particularly for C12:0, which may require facilitated transport across the enterocyte membrane (Wieland et al., 1993). Our results support the hypothesis of a positive synergistic interaction in FA utilization, when PUFA or MCFA are present, as both may facilitate the incorporation of less soluble FA through complementary mechanisms. Overall, these findings support the use of the (UFA + MCFA)/LCSFA ratio as a practical indicator of fat utilization efficiency in poultry nutrition. This aligns with the view of Wiseman and Blanch (1994), who argued that FA with fewer than 14 carbon atoms behave similarly to unsaturated ones in terms of absorption. In this study, the highest digestibility coefficients were observed in diets containing fat sources with a high (UFA + MCFA)/LCSFA ratio, such as SO (5.2), BSFO (2.4), and PKO (2.3). In contrast, PKFAD, characterized by the lowest (UFA + MCFA)/LCSFA ratio (1.6) together with a high LCSFA content (38.2%) and FFA level (68.2%), showed the poorest FA digestibility values among all treatments.

The high FFA content of the PKFAD diet resulted in a clearly detrimental effect on fat digestion. Broilers fed with this by-product showed lower digestibility of TFA, MUFA, and PUFA in both the jejunum and ileum compared to those fed SO, accompanied by reduced LCSFA absorption at the ileal level that approached significance. When compared to PKO, a fat source with a similar FA profile but lower FFA content, PKFAD also led to reduced FA digestibility, especially for MCFA and to a lesser extent for LCSFA. Supporting this interpretation, monoacylglycerols present in the duodenum are considered essential for the formation of mixed micelles and, consequently, for the absorption of lipid digestion products (Sklan, 1979; Krogdahl, 1985; Leeson and Atteh, 1995; Ravindran et al., 2016). Whereas previous studies indicated that dietary FFA negatively affect LCSFA absorption in young birds, likely due to the formation of insoluble soaps with calcium that reduce both fat and mineral availability (Small, 1991; Wiseman et al., 1991; Leeson and Atteh, 1995; Rodriguez-Sanchez et al., 2019b; Jimenez-Moya et al., 2021a), our findings suggest that MCFA absorption may also be impaired when FFA levels are elevated with greater impact observed as chain length increases. These limitations might be exacerbated in starter broilers due to their immature digestive system, including reduced bile salt secretion, incomplete micelle recycling, and limited monoacylglycerol availability (Leeson and Atteh, 1995; Tancharoenrat et al., 2014; Ravindran et al., 2016; Rodriguez-Sanchez et al., 2019a). These findings support the idea that elevated dietary FFA levels can impair the high FA absorption demonstrated in crude MCFA-rich fats, particularly in early developmental stages.

The apparent total tract digestibility confirms that fat absorption occurs primarily in the small intestine. Beyond the distal ileum, only minor increases in digestibility were observed, approximately 3% in TFA, likely due to microbial activity, which may lead to an overestimation of absorption when using excreta-based values, as these can also be influenced by endogenous FA losses (Lan et al., 2005). Consequently, apparent ileal digestibility is considered a more accurate indicator of true absorption than total tract digestibility, which may overestimate values due to post-ileal transformations (Rodriguez-Sanchez et al., 2019b).

The results of the present study align with several studies that have demonstrated that the jejunum is the primary site for TFA absorption in broilers, with reported contributions of 68% and 75% in young chickens fed unsaturated diets (Tancharoenrat et al., 2014; Rodriguez-Sanchez et al., 2019a, 2019b). The absorption dynamics along the intestinal tract indicate that it is influenced by the degree of carbon chain length, unsaturation, and FFA content. In this study, the absorption of MCFA and PUFA was very rapid and efficient, reaching around 92% in all diets at the jejunum level, whereas the ileum showed a contribution less than 8%. In contrast, the contribution of the ileum to the absorption of LCSFA and MUFA ranged from 9 to 26%, depending on the type of fat added. Compared to PKO, broilers fed BSFO showed a delayed uptake of LCSFA and MUFA, with compensatory absorption in the ileum reflected by an increase of 13% to 15% (*P* < 0.001), reaching apparent ileal digestibility values similar to those observed in birds fed PKO and SO. This pattern may be explained by the higher C16:0 content (approximately 7% more), along with lower levels of MCFA (13% less) and C14:0 (8.0% less) in BSFO compared to PKO. This highlights the role of the ileum in supporting FA absorption when their uptake is delayed in the upper intestinal segments.

### Histomorphology of jejunum and ileum

The effect of MCFA on intestinal morphology was assessed through villus height (Vh) and crypt depth (Cd), as these are key indicators of intestinal health and functionality, reflecting nutrient assimilation capacity (Wang and Peng, 2008). Longer villi are associated with a larger luminal absorptive area, leading to higher digestive enzyme activity and nutrient transport (Laudadio et al., 2012). The crypt is the region where stem cells divide and migrate upwards to form the villus, and its depth has been linked to the rate of villus renewal; the shallower the crypt, the lower the renewal rate (Oliveira et al., 2008). Across all treatments, Vh was higher in the jejunum than in the ileum, consistent with findings by Biasato et al. (2018). The progressive decrease in Vh along the small intestine has been attributed to differences in functionality and nutrient absorption among intestinal segments (Iji et al., 2001; de Verdal et al., 2010). Considering that MCFA serve as direct energy sources for enterocytes (Zentek et al., 2011), it could be expected that MCFA-rich fats (BSFO: 36.4%, PKO: 49.0%) would positively influence intestinal morphology compared to soybean oil, which lacks MCFA. However, in the present study, no significant differences were observed in histomorphological variables (Vh, Cd, and Vh/Cd) in either the jejunum or ileum of broilers fed BSFO and PKO diets compared to those receiving soybean oil. Nevertheless, villus height in the jejunum approached significance (*P* = 0.069), with lower values in broilers fed the PKFAD diet compared with the other treatments, suggesting that high FFA levels may have negatively affected intestinal morphology. Similar results were reported by Zeitz et al. (2015) in broilers fed diets rich in esterified lauric acid or a control diet high in oleic and linoleic acids esterified to TAG. In contrast, an FFA effect on ileal Vh was observed between broilers fed PKO and PKFAD diets, which contained similar MCFA levels (32.5%) but differed in FFA content (PKO: 8.1% vs. PKFAD: 66.1%). The lower Vh values in broilers fed PKFAD diets suggest a potential negative effect of high FFA levels, as proposed by Palomar et al. (2023) and Zeitz et al. (2015), possibly impairing absorptive capacity in the ileum. This finding may be associated with the reduced TFA digestibility observed in broilers fed PKFAD diets compared to those fed PKO diets (*P* = 0.042), likely related to the lower digestibility of LCSFA in this intestinal segment, and consistent with their reduced growth performance.

### Microbiological counting

A stable intestinal microbiota plays a key role in nutrient absorption, immune regulation, and overall health in broiler chickens (Liu et al., 2021), and classical bacterial indicators are often used as complementary functional markers to assess gut functionality. In the present study, diets supplemented with MCFA-rich fats (BSFO, PKO, and PKFAD) supported microbial stability in the ileum of young broilers, with no significant alterations compared to the SO group (*P* > 0.05). However, higher Enterobacteriaceae, total coliform, and Escherichia coli counts approached significance in MCFA-rich-fed birds (*P* ≤ 0.070; Table 7). These findings do not support previous studies reporting antimicrobial effects of MCFA-rich sources, including reductions in pathogenic bacteria in the jejunum with BSFO (Kierończyk et al., 2022; Dewanti et al., 2024; Dabbou et al., 2025), as well as the antimicrobial activity attributed to PKO and its refining by-product, PKFAD (Izuddin et al., 2023). This discrepancy may be attributed to the rapid absorption of MCFA in the upper intestinal tract, resulting in low concentrations reaching distal intestinal sections and thereby limiting their antimicrobial effects. Overall, these results indicate that replacing SO with MCFA-rich fat sources did not markedly alter ileal microbial populations, supporting their use as functional alternatives to conventional oils.

### Cecal VFA content

The gastrointestinal tract of broilers hosts a diverse and dynamic microbial community (Gong et al., 2019). The interactions between these microorganisms, the SCFA they produce, and intestinal morphology are particularly relevant in the ileum and cecum (Sekelja et al., 2012). In the cecum, microbial fermentation generates SCFA such as acetate (C2:0), propionate (C3:0), and butyrate (C4:0), which are key metabolites involved in gut health (Józefiak et al., 2004; Dauksiene et al., 2021; Liu et al., 2021). These compounds have been associated with improved intestinal structure, immune modulation, and pathogen inhibition in poultry (Liou et al., 2013; Liu et al., 2021). In the present study, broilers fed crude oils rich in MCFA (BSFO and PKO) showed higher cecal propionate concentrations compared to those receiving the FFA-rich diet (PKFAD). Increased propionate has been associated with a higher abundance of *Bacteroidetes spp*., which produce this SCFA via the succinate pathway (Liou et al., 2013; Dauksiene et al., 2021). Although research on MCFA-rich oils and their impact on the broiler microbiota remains limited, Dauksiene et al. (2021) reported increased cecal propionate following dietary inclusion of MCFA (C6–C12) at 0.2%, suggesting a modulatory effect on bacterial activity. These findings support the hypothesis that high MCFA levels and low FFA concentrations may contribute to microbial stability and ecological modulation along the gastrointestinal tract. Conversely, the lower propionate concentrations observed in birds fed the PKFAD diet may be explained by the elevated levels of FFA, which can negatively affect the gut environment by disrupting microbial activity and fermentation processes (Baltić et al., 2018; Moran, 2021). Whereas SCFA are microbial fermentation products with established roles in gut health, the contribution of MCFA to microbial modulation remains less explored, partly because MCFA are rapidly absorbed and not derived from fermentation. Nevertheless, fermentation outcomes depend on a mature and functional microbiota (Gomez-Osorio et al., 2021), and the full establishment of cecal flora may require up to 30 days (Amit-Romach et al., 2004). To further investigate these microbial responses and SCFA dynamics, the application of 16S rRNA sequencing remains a valuable tool for detailed characterization of the cecal microbiome.

In conclusion, this study supports the use of MCFA-rich fat sources as functional alternatives to conventional oils in broiler starter diets, with regards to fat digestibility and growth performance. The MCFA demonstrated high ileal digestibility, with values reaching up to 89%, particularly for lauric acid (C12:0), whose rapid absorption occurred predominantly in the jejunum, contributing up to 92.04% of total uptake. In young broilers, the efficiency of MCFA utilization was comparable to that of PUFA and clearly superior to that of LCSFA such as palmitic and stearic acids. However, elevated dietary FFA levels limited MCFA absorption and negatively affected AME intake and growth performance, as observed in birds fed the PKFAD diet. In contrast, inclusion of 4% crude oils rich in MCFA, particularly C12:0, and low in FFA supported efficient fat absorption while maintaining growth performance and intestinal health. Although no major differences were observed in most intestinal morphology variables or general microbial counts, a reduction in ileal villus height was detected in broilers fed the FFA-rich PKFAD diet compared to those receiving PKO. Broilers fed BSFO also showed increased cecal propionate concentrations, indicating a potential fermentative response with metabolic implications.

Taken together, these findings reinforce the suitability of BSFO and PKO as viable dietary fat sources in early broiler nutrition, with their high digestibility, functional benefits, and origin as agro-industrial co-products aligning with circular and resource-efficient strategies for sustainable poultry production. At the same time, the results highlight the critical importance of fat quality, although co-products with excessive levels of FFA, such as PKFAD, may compromise feed intake and lipid digestibility.

## CRediT authorship contribution statement

**M. Espinosa-de-los-Monteros-Peñafiel:** Writing – review & editing, Writing – original draft, Visualization, Methodology, Investigation, Formal analysis, Data curation. **L. Castillejos:** Writing – review & editing, Visualization, Supervision, Resources, Methodology, Investigation, Formal analysis, Data curation, Conceptualization. **R. Sala:** Writing – review & editing, Visualization, Supervision, Resources, Project administration, Methodology, Investigation, Funding acquisition, Conceptualization. **A. Tres:** Writing – review & editing, Methodology, Formal analysis, Data curation. **M.D. Soler:** Writing – review & editing, Methodology, Formal analysis, Data curation. **A.C. Barroeta:** Writing – review & editing, Visualization, Supervision, Resources, Project administration, Methodology, Investigation, Funding acquisition, Formal analysis, Data curation, Conceptualization.

## Disclosures

The authors declare the following financial interests/personal relationships which may be considered as potential competing interests:

Ana Cristina Barroeta reports financial support and administrative support were provided by Spain Ministry of Science Innovation and Universities. If there are other authors, they declare that they have no known competing financial interests or personal relationships that could have appeared to influence the work reported in this paper.
